# A Review of Outcome Data concerning Children Born following Assisted Reproductive Technologies

**DOI:** 10.5402/2012/405382

**Published:** 2012-06-17

**Authors:** Charlotte Dupont, Christophe Sifer

**Affiliations:** ^1^Service d'Histologie-Embryologie-Cytogénétique, Laboratoire de Biologie de la Reproduction, Centre Hospitalier Universitaire Jean Verdier, Assistance Publique-Hôpitaux de Paris, 93140 Bondy, France; ^2^Service d'Histologie-Embryologie-Cytogénétique, Hôpital Jean Verdier, 93140 Bondy, France

## Abstract

Assisted reproductive technologies (ARTS) are used for more than 30 years to help infertile couples. Concerns about long-term health of children conceived following ART have led to start follow-up studies. Despite methodological limitations and discrepant results, many of the studies and meta-analyses have reported an increased risk of birth defects after ART. Etiologies may be multiple births, a major drawback of ART, parents' subfertility, or technologies themselves. Prematurity and intrauterine growth retardation (IUGR) seem to cause most of the pathologies reported in ART children. Nevertheless, epigenetic disorders need to be followed up since increases of imprinting diseases were reported. Consequently, alteration of gametes and early embryo development with ART may have consequences on children health since periconceptional period is critical for long-term development. Yet general condition of most of children conceived with ART is reassuring, but long-term followup is still strongly needed.

## 1. Introduction

Currently, more than 3 500 000 children were born following assisted reproductive technologies (ARTS) [1]. It means that, in developed countries, 0.8 to 4.1% of children were conceived after ART [2].

Since Louise Brown was born in 1978, *in vitro* fertilization (IVF) has been used commonly. Despite many concerns about long-term effects of this technology, very few follow-up studies of these children were attempted.

In 1992, a new technology, intracytoplasmic sperm injection (ICSI), had been developed and had spread throughout the world [3]. Since a spermatozoon is injected through the oocyte membrane, this technology was considered more invasive and many followups of children born after ICSI were designed. In a same time, more surveys assessing health of children born after conventional IVF were started. 

Other technologies have emerged like embryo cryopreservation with slow freezing protocols and more recently vitrification. Preimplantation genetic diagnosis (PGD) or screening (PGS) used to detect and eliminate embryos with single-gene disorders or aneuploidy is quite invasive and demand more embryo handling and may be at risk of altered embryo and fetal developments. 

Concerns about long-term health of people conceived with ART still remain, and many studies have been conducted worldwide. Despite methodological limitations, different designs of these studies and other bias may have led to contradictory results, and many of the studies and meta-analyses reported from the followup of children born after ART concluded an increased risk of birth defects.

## 2. Risks for Children Born after ART

### 2.1. Birth Defects following ART

#### 2.1.1. Sequence of Pregnancy

Multiple pregnancies are a major drawback of ART, leading to serious obstetrical risks. After spontaneous conception, multiple pregnancy risk is about 1% whereas it is about 25% following ART. Higher risks of prematurity, low birth weight, perinatal death, and neurologic sequelae (learning disability, language problems, etc.) have been reported concerning multiple births. To avoid such drawbacks, a policy of elective single embryo transfer (eSET) has been developed by many countries [4].

Even if multiple pregnancy risks are well known, it was also highlighted that pregnancy risks are more frequent in singleton conceived with ART. Data reported in 3 meta analyses have demonstrated that singletons born following ART (IVF, ICSI, GIFT) have twofold risks of low or very low birth weight, prematurity, and intrauterine growth retardation (IUGR) compared to singleton conceived spontaneously [5–7]. Higher risks of perinatal and neonatal death and more admission into intensive care units were observed regarding from ART children [5].

Furthermore, a link between time to conceive (as an index of subfertility of the parents) and risk of prematurity and low birth weight has been established [8].

In a multicentre study conducted on 1 515 five-year-old children, Bonduelle et al. showed an increased risk of major and minor malformations detected from the neonatal period up to 5 years [9]. Incidence of urogenital malformations was increased in ICSI boys leading to genitourinary surgery. Furthermore, children conceived with ART were less mature at birth and seem to have more childhood illness, admission to hospital, and surgery. Nevertheless, no difference concerning either the height or weight of 5-year-old ART children was observed compared to general population [10]. The conclusion of this study concerning health of children conceived with IVF or ICSI was not alarming, but it highlights the risk of increased childhood illness that needs to be monitored [10].

No different temperaments and behaviour issues were observed in IVF or ICSI children compared to naturally conceived children. However, psychological testing on parents has underlined that mother who conceived child with ICSI seems to have a more commitment to parenting [11].

#### 2.1.2. Chromosomal Abnormalities

Several reports have observed, studying subfertile couples, a small increase of chromosomal abnormalities especially in males with deep infertility [12]. Abnormal karyotype was detected in 13,7% of azoospermic men and 4,6% of oligozoospermic men [13]. Consequently, male infertility is associated with higher chromosomal abnormalities in spermatozoa leading to more chromosomal abnormalities in children born following ICSI [9]. Bonduelle has observed more prenatal abnormalities (2,1%) when spermatozoon is derived from men whose sperm concentration was below 20 M/mL compared to those with concentration superior to 20 M/mL [9]. 

### 2.2. Hotspots relating with Birth Defects in ART

Many steps are needed to achieve pregnancy with ART. All the stages are critical and may influence long-term health of the conceptus.

#### 2.2.1. Hormonal Stimulation and Oocyte Environment

Induction of ovulation with high doses of gonadotrophin alters oocyte maturation process and creates an artificial environment in oviduct and uterus, which can influence fertilisation and preimplantation embryos.

#### 2.2.2. Embryo Culture

With IVF success, many concerns about embryo culture appeared. Embryos developed *in vitro* are submitted to altered hormonal environment and exogenous chemical compounds. Since 1993, thanks to animal models, it was obvious that embryo culture could impact on fetal development and expression of imprinted genes [14]. Furthermore, a study comparing two embryo culture mediums in human IVF demonstrated that different medium compositions could affect birth weight in singleton [15].

#### 2.2.3. ICSI

When ICSI was started, many risks were highlighted. First of all, it was obvious that there were more chromosomal abnormalities and gene disorders in spermatozoa used for ICSI. These defects can impact on embryo development, and chromosomal abnormality can be transmitted in case of sperm aneuploidy. Offspring run a risk of gene abnormalities related to fertility issue inheritance [9].

Furthermore, when ICSI is performed, a spermatozoon is injected directly into the cytoplasm of each oocyte. Then, first steps of fertilization are bypassed leading to a loss of selection of morphologically normal spermatozoon, even if data on this specific topic showed that there is no selection of spermatozoon in spontaneous conception [16].

Finally, even if the injection of spermatozoon with chromosomal abnormalities is the most probable cause of higher incidence of chromosomal abnormalities in fetus, risk may be linked to the process of ICSI itself. The breaking of the zona pellucida and cytoplasmic membrane could lead to injuries of internal structures of the oocyte and have deleterious consequences such as aneuploidy and chromosomal abnormalities [9].

#### 2.2.4. Cryopreservation

Few followups of children conceived with cryopreserved embryo transfer are available. However, more major malformations were observed in ICSI children born after cryopreserved embryo transfer. Furthermore, children conceived with cryopreserved embryos tended to have more chromosomal abnormalities and have significant higher birthweight than children conceived with fresh embryos [17]. It was demonstrated in mice that cryopreservation of preimplantation embryo may have small long-term consequences such as increased body weight and different behaviour [18].

However, Wennerholm et al. published a recent review concerning the long-term outcome of children conceived with cryopreserved embryo transfer. Their results were reassuring despite an increasing risk of low birthweight and prematurity following the slow freezing method, and they conclude that data on children conceived with embryo cryopreserved with vitrification are needed [19].

### 2.3. Imprinted Genes and Epigenetic

#### 2.3.1. Epigenetic and Development

Germ cell and preimplantation embryo developments are critical stages for epigenetic events. Indeed, DNA methylation is erased in primordial germ cells and reapposed during spermatozoon and oocyte maturation in a sex-specific manner. After fertilization, the epigenetic resetting of the gamete genome leads to the activation of developmental genes and allows pluripotency, but imprinting genes are protected from demethylation. Imprinting genes are functionally haploid and are expressed in a parent-of-origin-specific manner (expression from a single parental allele). To date, 100 imprinting genes are known and play a critical role in embryo development and fetus growth.

Epigenetic disruptions may take root during gametogenesis, fertilization, and preimplantation embryo development. Gamete and embryo exposure to artificial conditions may lead to epimutation and altered imprinting genes with consequences on fetal development.

#### 2.3.2. ART and Epigenetic

In human sperm, altered methylations of H19 DMR (differentially methylated regions) were observed in patients with oligozoospermia, teratozoospermia, or oligoasthenoteratozoospermia [20, 21].

It was demonstrated in mouse and human that ovarian stimulation may alter methylation of imprinting genes. Despite H19 is a maternally expressed gene only methylated on the paternal chromosome, methylation was found in H19 DMRs of superovulated mouse oocyte and of human superovulated immature oocytes [22]. Fauque et al. also demonstrated an altered expression of H19 in superovulated mouse oocytes [23]. These results emphasize the risk of epimutation caused by ovarian stimulation. Furthermore, embryo culture mediums also have impact on genomic imprinting. It was previously shown that IVF and *in vitro *culture have an effect on DNA methylation [23]. Furthermore, Doherty et al. demonstrated in mice that embryo culture in Whitten's medium leads to loss of methylation of paternal allele of H19 and results in biallelic expression [24]. In mice, Khosla et al. have compared *in vitro* embryo culture and *in vivo *embryo development. They demonstrated that serum in culture medium affects postimplantation embryo development. Fetuses were lighter, and expression of the imprinted H19 and Igf2 genes was decreased [25]. 

Consequently, culture medium composition can affect the expression of imprinted genes and influence phenotype of the conceptus.

The mechanisms of epimutations are still unknown.

#### 2.3.3. ART and Imprinting Diseases

A link between imprinting disorders and ART is known for a few years [26]. It was observed a higher proportion of ART conceptions among children with Beckwith-Wiedemann syndrome (BWS). It was reported more than 60 patients suffering from BWS and conceived with ART [27]. Hypomethylation of one of the two maternal DMR at chromosome 11p15 was the most frequent mechanism observed in these patients [26]. BWS is characterized by increased fetal and neonatal growth, macroglossia, neonatal hypoglycaemia, and risk of embryonal tumors (Wilms tumor). Connection between BWS and “large offspring syndrome” characterized by an overgrowth syndrome and epigenetic alterations observed in cattle conceived with IVF may be done [14]. 

An association between Angelman syndrome (AS) and ICSI was also observed [26, 27], and one patient with AS caused by epimutation and conceived after ovarian hyperstimulation was reported [28]. AS is characterised by neurogenetic alterations with developmental delay, intellectual disability and may results from imprinting defect leading to loss of function of UBE3A gene carried by maternal chromosome 15. Furthermore, higher risks of AS caused by epegentic defects have been highlighted in patients born from subfertile couple [28]. However, this syndrome is rare, and the theorical relative risk to develop AS after ART appears to be low. 

Epigenetic disorders were identified in majority of cases of AS and BWS after ART, and defect of maternal allele methylation was detected in all cases of AS and BWS caused by epigenetic disorders [27]. Consequently, there are enough arguments to suggest an increased risk of imprinting disorders caused by ART.

Very few data are available to link ART with other imprinting diseases such as Prader-Willi syndrome and Russel-Silver syndrome, and more epidemiological studies are needed.

Nevertheless, some other large epidemiological studies [29–31] did not show any increase of epigenetic disorders in children conceived with ART. 

### 2.4. ART and Other Pathologies

#### 2.4.1. ART and Cancer

Many studies have highlighted an increased risk of cancer in children born following IVF-ICSI [32], but some contradictory results lead to reconsider this hypothesis [33]. Increasing cancer in ART children may be linked more indirectly to altered neonatal development following such procedures [34]. 

Furthermore, long-term studies would be necessary to highlight an increased risk of adult cancer.

#### 2.4.2. ART and Neurological Outcomes

Discrepant data are available on this topic and have underlined that neurological abnormalities and cerebral palsy may be more frequent or not in ARTchildren. A recent systematic review taking account of methodological bias concluded that children conceived after IVF/ICSI do not have impaired neuromotor, cognitive, language, and behavioural development compared to naturally conceived children [35]. In this study, the observed neurological effects may be consequences of prematurity and low birth weight observed in ART children especially in case of multiple births.

#### 2.4.3. ART and Cardiovascular Diseases

Very few data concerning cardiovascular outcomes of ART children are available and are strongly needed. In 2007, Painter and Roseboom emphasized the lack of information about this subject [36]. As it was observed increased risks of low or very low birth weight, prematurity, and IUGR in ART children, they should be at risks of metabolic syndrome in adulthood. Indeed, in the nineties, an epidemiological study underlined an increased risk of noninherited metabolic diseases in people born small for gestational age (SGA) [37]. Metabolic syndrome characterised by obesity, type 2 diabetes (T2D), and hypertension may take root during early development, throughout gestation as stated in the “Developmental Origins of Health and Disease” (DOHaD) hypothesis [38]. Studies including adult born following IVF or ICSI are needed to assess metabolic syndrome occurrence. Nevertheless, in a recent study, Ceelen et al. have observed an increased blood pressure levels in IVF children compared to naturally conceived children. However, blood pressures levels were not correlated to birthweight or body size [39].

## 3. Methodological Limitations

Followup of ART children should be proceeded by all ART centres but is time-consuming, and researchers are confronted by methodological difficulties. Indeed, researchers need to contact families many years after birth and can create a risk of stigmatization of ART children. High rate of nonparticipation is reported in many studies, depending on cultural influences and legal rules of each country that could generate a bias of selection because of numerous refusal or lost to followup [10]. Furthermore, it may be difficult to design a control group, and sample size needs to be adequate. ARTchildren stay more often in neonatal unit care compared to naturally conceived children and are checked very carefully leading to overestimation of anomalies that create an observational bias. Definitions of major or minor malformations are different between studies. The start of children examination and duration of the followup vary from birth to adolescence according to studies. 

## 4. Conclusion and Perspectives

There are clear evidences of birth defects after ART. Periconceptional and prenatal periods are critical for long-term development. Several etiologies may involve parents' subfertility or technologies. To date, multiple births represent the major risk of ART defects, and, reducing the number of embryo transferred to minimize, this drawback should be a strong priority. ART singletons are also at risk of impaired development since slightly increase of childhood illness and more hospital admission are reported. Nevertheless, prematurity and IUGR seem to be causes of most of the pathologies reported in ART children. On the other hand, researchers need to continue paying attention of imprinting diseases. Finally, general condition of most of children conceived with ART is reassuring, but parents undergoing ART should be aware of such existing risks. Long-term followups are still strongly needed.

Currently, first IVF people are now more than 30 years old, and some of them have conceived children. We can wonder if children born from parents conceived following ART are at risk of altered development. Indeed, epidemiological studies on nutrition and fetal programming have suggested that exposure of paternal grandfathers to famine influences obesity and cardiovascular disease in subsequent generations [40, 41]. These results emphasize the possibility of programming further generations. Will the same observations be made with ART?

## References

[1] Zegers-Hochschild F, Adamson GD, de Mouzon J (2009). The International Committee for Monitoring Assisted Reproductive Technology (ICMART) and the World Health Organization (WHO) revised glossary on ART terminology, 2009. *Human Reproduction*.

[2] De Mouzon J, Goossens V, Bhattacharya S (2010). Assisted reproductive technology in Europe, 2006: results generated from European registers by ESHRE. *Human Reproduction*.

[3] Palermo G, Joris H, Devroey P, Van Steirteghem AC (1992). Pregnancies after intracytoplasmic injection of single spermatozoon into an oocyte. *The Lancet*.

[4] Ombelet W, De Sutter P, Van der Elst J, Martens G (2005). Multiple gestation and infertility treatment: registration, reflection and reaction—the Belgian project. *Human Reproduction Update*.

[5] Hansen M, Bower C, Milne E, de Klerk N, Kurinczuk JJ (2005). Assisted reproductive technologies and the risk of birth defects—a systematic review. *Human Reproduction*.

[6] Jackson RA, Gibson KA, Wu YW, Croughan MS (2004). Perinatal outcomes in singletons following in vitro fertilization: a meta-analysis. *Obstetrics and Gynecology*.

[7] McGovern PG, Llorens AJ, Skurnick JH, Weiss G, Goldsmith LT (2004). Increased risk of preterm birth in singleton pregnancies resulting from in vitro fertilization-embryo transfer or gamete intrafallopian transfer: a meta-analysis. *Fertility and Sterility*.

[8] Ghazi HA, Spielberger C, Kallen B (1991). Delivery outcome after infertility—a registry study. *Fertility and Sterility*.

[9] Bonduelle M, Van Assche E, Joris H (2002). Prenatal testing in ICSI pregnancies: Incidence of chromosomal anomalies in 1586 karyotypes and relation to sperm parameters. *Human Reproduction*.

[10] Bonduelle M, Wennerholm UB, Loft A (2005). A multi-centre cohort study of the physical health of 5-year-old children conceived after intracytoplasmic sperm injection, in vitro fertilization and natural conception. *Human Reproduction*.

[11] Barnes J, Sutcliffe AG, Kristoffersen I (2004). The influence of assisted reproduction on family functioning and children’s socio-emotional development: results from a European study. *Human Reproduction*.

[12] Schreurs A, Legius E, Meuleman C, Fryns JP, D’Hooghe TM (2000). Increased frequency of chromosomal abnormalities in female partners of couples undergoing in vitro fertilization or intracytoplasmic sperm injection. *Fertility and Sterility*.

[13] Van Assche E, Bonduelle M, Tournaye H (1996). Cytogenetics of infertile men. *Human Reproduction*.

[14] Young LE, Fernandes K, McEvoy TG (2001). Epigenetic change in IGF2R is associated with fetal overgrowth after sheep embryo culture. *Nature Genetics*.

[15] Dumoulin JC, Land JA, Van Montfoort AP (2010). Effect of in vitro culture of human embryos on birthweight of newborns. *Human Reproduction*.

[16] Sakkas D, Moffatt O, Manicardi GC, Mariethoz E, Tarozzi N, Bizzaro D (2002). Nature of DNA damage in ejaculated human spermatozoa and the possible involvement of apoptosis. *Biology of Reproduction*.

[17] Belva F, Henriet S, van den Abbeel E (2008). Neonatal outcome of 937 children born after transfer of cryopreserved embryos obtained by ICSI and IVF and comparison with outcome data of fresh ICSI and IVF cycles. *Human Reproduction*.

[18] Dulioust E, Toyama K, Busnel MC (1995). Long-term effects of embryo freezing in mice. *Proceedings of the National Academy of Sciences of the United States of America*.

[19] Wennerholm UB, Söderström-Anttila V, Bergh C (2009). Children born after cryopreservation of embryos or oocytes: a systematic review of outcome data. *Human Reproduction*.

[20] Boissonnas CC, Abdalaoui HE, Haelewyn V (2010). Specific epigenetic alterations of IGF2-H19 locus in spermatozoa from infertile men. *European Journal of Human Genetics*.

[21] Marques CJ, Carvalho F, Sousa M, Barros A (2004). Genomic imprinting in disruptive spermatogenesis. *The Lancet*.

[22] Sato A, Otsu E, Negishi H, Utsunomiya T, Arima T (2007). Aberrant DNA methylation of imprinted loci in superovulated oocytes. *Human Reproduction*.

[23] Fauque P, Jouannet P, Lesaffre C (2007). Assisted reproductive technology affects developmental kinetics, H19 imprinting control region methylation and H19 gene expression in individual mouse embryos. *BMC Developmental Biology*.

[24] Doherty AS, Mann MRW, Tremblay KD, Bartolomei MS, Schultz RM (2000). Differential effects of culture on imprinted H19 expression in the preimplantation mouse embryo. *Biology of Reproduction*.

[25] Khosla S, Dean W, Brown D, Reik W, Feil R (2001). Culture of preimplantation mouse embryos affects fetal development and the expression of imprinted genes. *Biology of Reproduction*.

[26] Amor DJ, Halliday J (2008). A review of known imprinting syndromes and their association with assisted reproduction technologies. *Human Reproduction*.

[27] Gosden R, Trasler J, Lucifero D, Faddy M (2003). Rare congenital disorders, imprinted genes, and assisted reproductive technology. *The Lancet*.

[28] Ludwig M, Katalinic A, Groß S, Sutcliffe A, Varon R, Horsthemke B (2005). Increased prevalence of imprinting defects in patients with Angelman syndrome born to subfertile couples. *Journal of Medical Genetics*.

[29] Bowdin S, Allen C, Kirby G (2007). A survey of assisted reproductive technology births and imprinting disorders. *Human Reproduction*.

[30] Lidegaard O, Pinborgand A, Andersen N (2006). Imprinting disorders after assisted reproductive technologies. *Current Opinion in Obstetrics and Gynecology*.

[31] Sutcliffe AG, Peters CJ, Bowdin S (2006). Assisted reproductive therapies and imprinting disorders—a preliminary British survey. *Human Reproduction*.

[32] Kallen B, Finnstrom O, Lindam A, Nilsson E, Nygren KG, Olausson PO (2010). Cancer risk in children and young adults conceived by in vitro fertilization. *Pediatrics*.

[33] Bruinsma F, Venn A, Lancaster P, Speirs A, Healy D (2000). Incidence of cancer in children born after in-vitro fertilization. *Human Reproduction*.

[34] Savage T, Peek J, Hofman PL, Cutfield WS (2011). Childhood outcomes of assisted reproductive technology. *Human Reproduction*.

[35] Middelburg KJ, Heineman MJ, Bos AF, Hadders-Algra M (2008). Neuromotor, cognitive, language and behavioural outcome in children born following IVF or ICSI—a systematic review. *Human Reproduction Update*.

[36] Painter RC, Roseboom TJ (2007). Cardiovascular health among children born after assisted reproduction. *European Journal of Obstetrics & Gynecology and Reproductive Biology*.

[37] Barker DJP, Eriksson JG, Forsén T, Osmond C (2002). Fetal origins of adult disease: strength of effects and biological basis. *International Journal of Epidemiology*.

[38] Gluckman PD, Hanson MA (2004). The developmental origins of the metabolic syndrome. *Trends in Endocrinology & Metabolism*.

[39] Ceelen M, van Weissenbruch MM, Vermeiden JPW (2008). Cardiometabolic differences in children born after in vitro fertilization: follow-up study. *Journal of Clinical Endocrinology and Metabolism*.

[40] Kaati G, Bygren LO, Edvinson S (2002). Cardiovascular and diabetes mortality determined by nutrition during parents’ and grandparents’ slow growth period. *European Journal of Human Genetics*.

[41] Kaati G, Bygren LO, Pembrey M, Sjostrom M (2007). Transgenerational response to nutrition, early life circumstances and longevity. *European Journal of Human Genetics*.

